# *Lycium barbarum* Reduces Abdominal Fat and Improves Lipid Profile and Antioxidant Status in Patients with Metabolic Syndrome

**DOI:** 10.1155/2017/9763210

**Published:** 2017-06-08

**Authors:** Mayara Zagonel de Souza Zanchet, Geisson Marcos Nardi, Letícia de Oliveira Souza Bratti, Fabíola Branco Filippin-Monteiro, Claudriana Locatelli

**Affiliations:** ^1^Programa de Pós Graduação em Biociências e Saúde, Universidade do Oeste de Santa Catarina (UNOESC), Joaçaba, SC, Brazil; ^2^Curso de Medicina, Instituto de Ciências Exatas e Naturais (ICEN), Universidade Federal de Mato Grosso (UFMT), Rondonópolis, MT, Brazil; ^3^Departamento de Análises Clínicas, Universidade Federal de Santa Catarina (UFSC), Florianópolis, SC, Brazil; ^4^Laboratório de Bioquimica Experimental, Universidade do Oeste de Santa Catarina (UNOESC), Joaçaba, SC, Brazil

## Abstract

Natural antioxidants present in fruits have attracted considerable interest due to their presumed safety and potential nutritional value. Even though antioxidant activities of many fruits have been reported, the effects of phytochemicals of goji berry (GB) in patients with metabolic syndrome have not been investigated. In this study, we examined anthropometric and biochemical parameters in patients with metabolic syndrome after the consumption of GB. The patients were divided into two groups, control (C) and supplemented (S), and followed up for 45 days. Participants were individually instructed to carry out a healthy diet, but additionally, an inclusion of 14 g of the natural form of goji berry in the diet during 45 days for the S group was proposed. After 45 days of study, a significant reduction in transaminases as well as an improvement in lipid profile in the S group was observed. Likewise, a significant reduction in the waist circumference of the S group was observed when compared with that of the C group, and increased glutathione and catalase levels associated with a reduction of lipid peroxidation. These results suggest that this is an effective dietary supplement for the prevention of cardiovascular diseases in individuals with metabolic syndrome.

## 1. Introduction

Metabolic syndrome (MS) consists of different risk factors for cardiovascular disease (CVD) and diabetes mellitus type 2 (DM2) [1], and it includes a cluster of metabolic abnormalities such as abdominal obesity, dyslipidemia, hyperglycemia, and hypertension, leading to an increase in the levels of oxidative stress, concomitantly reducing antioxidant defenses [2, 3]. It is one of the main clinical challenges in public health, being considered a group of risk factors for cardiovascular disease, due to the accumulation of abdominal fat and the increase in inflammatory mediators and oxidative stress [3].

Oxidative stress has been implicated in the development and progression of many diseases such as atherosclerosis, inflammation, cancer, neurodegenerative diseases, and diabetes [4–6]. Redox homeostasis, being a metabolic equilibrium between reduction and oxidation, is important in maintaining normal metabolism by ensuring proper response from the cells to either endogenous or exogenous stimuli. Energy harvesting through cellular redox process releases by-products as reactive species: oxygen (ROS) and nitrogen (RNS). These reactive species are crucial for cell signaling. Overwhelming levels and dysregulation of the reactive species, however, disrupt the delicate balance [7]. The shift towards the oxidized state leads to oxidative stress which can be defined as an imbalance between endogenous antioxidant defense mechanisms and the production of ROS, which at high levels can cause cell injury and damage through modifications of proteins, lipids, and DNA [8, 9].

In order to reduce the deleterious effects of oxidative stress, several antioxidant protective networks and signaling pathways are operative in cells [10]. The most important enzymatic antioxidant system is composed of superoxide dismutase (SOD), catalase (CAT), and glutathione peroxidase (GPx), which protect the cells against damage caused by the radical superoxide and hydrogen peroxide. In addition to the enzymatic system, the cells have a nonenzymatic system, especially glutathione [8, 9].

Previous studies have demonstrated a close relationship between the complications associated with MS and subcutaneous adipose tissue, as well as the characteristics of the visceral adipose tissue. When the adipocyte homeostasis is altered due to excessive calorie intake, sedentarism, or genetic variants, among others, several inflammatory adipose tissue-derived cytokines are released, such as interleukin 1 beta (IL-1*β*), interleukin 18 (IL-18), interleukin 6 (IL-6), and tumor necrosis factor alpha (TNF-*α*), as well as induction of mononuclear leukocytes (lymphocytes and monocytes) [11]. Such cytokines constitute a well-stablished link between insulin resistance and endothelial dysfunction, a precursor of atherosclerosis, another hallmark of MS [12].

Epidemiological studies have found that consumption of fruits and vegetables has attracted growing interest because of their significant role in reducing the risk of cardiovascular diseases and other chronic diseases [13, 14]. Several studies demonstrated that medicinal plants and fruits are a rich source of antioxidant compounds such as phenolics, flavonoids, quinones, vitamins, and alkaloids, which can decrease the incidence of oxidative stress and associated diseases [15–17]. Recent studies have demonstrated the effects of polyphenolic compounds in improving clinical factors associated with MS. Epidemiological evidence shows that the consumption of food rich in polyphenolic compounds in Asian populations reduces cardiovascular risk factors. However, there are limited studies associating the consumption of polyphenolic compounds in western populations [16].

A negative correlation has been shown between MS and polyphenolic compound ingestion and vitamins A, C, and E [17]. The addition of flavonoids on a diet could be an effective strategy for MS prevention with possible improvements in blood pressure, systemic inflammation, and oxidative stress [18].

Natural antioxidants that are presented in fruits have attracted considerable interest because of their presumed safety and potential nutritional and therapeutic value. The increased interest in natural antioxidants has led to the antioxidant evaluation of many species of fruits [14].

Among the fruits with the highest amount of antioxidant substances is the *Lycium barbarum* also named goji berry (GB). *Lycium barbarum*, a traditional Chinese medicine plant, is a small red fruit widely consumed in China due to its benefits to vision, kidney, liver, and diseases such as diabetes mellitus and obesity [19]. It is considered the richest natural source of antioxidants vitamin C, flavonoids, and carotenoids especially zeaxanthin, quercetin, and rutin [20–22].

A number of preclinical studies and a few clinical studies on the pharmacological activities and possible mechanisms of GB have been reported in the literature. GB exhibits a wide array of therapeutic/medicinal effects on aging, fatigue, cancer, colitis, stroke, diabetes, Alzheimer's disease, hepatoprotection, immunomodulation, and glaucoma in different animal models [23–26].

Although the antioxidant benefits of GB have already been highlighted in several in vitro and in vivo studies, no previous study has shown the effect of GB supplementation *in natura* on MS patients. In this study, we hypothesize that the administration of GB in patients with MS could positively influence glucose homeostasis, lipid profile, hepatic markers, and biomarkers of inflammation and oxidative stress.

## 2. Material and Methods

### 2.1. Participants

This study was approved by the University of Alto Vale do Rio do Peixe, and free informed consent of all participants was obtained (CAAE 1,304,016/2015). The recruitment has been made during individualized clinical evaluation in the Basic Health Unit of Peritiba, Santa Catarina, Brazil. Patients were selected according to initial screening results based on biochemical and anthropometric measurements. All participants were included as MS patients when possessing at least 3 of the following characteristics: abdominal obesity (waist circumference ≥ 90 cm in men and ≥80 cm in women); hypertriglyceridemia (triglycerides (TG) > 150 mg/dl); low high-density lipoprotein (HDL-c < 40 mg/dl in men and <50 mg/dl in women); high low-density lipoprotein (LDL-C > 160 mg/dl); increased systolic blood pressure (SBP) ≥130 mmHg and diastolic blood pressure (DBP) ≥85 mmHg; and glycemia >100 mg/dl in men or women in adulthood or elderly stage between the ages of 32 and 76. Individuals using antidiabetic, antihypertensive, and lipid-lowering medications were considered with fasting glucose, high blood pressure, and dyslipidemia, respectively. Exclusion criteria included not being part of individualized care, not being diagnosed with MS, not following the treatment, and showing any hypersensitivity to GB usage. The study consisted of 50 patients, randomly divided into two groups, referred to as the control group (C) and supplemented group (S).

### 2.2. GB Intake Intervention

GB used in the study were purchased from Ningxia Toyo Trade Co., Ltd., China. Portions of GB were provided in a daily fractionated portions. All participants were individually oriented to carry out a healthy diet, based on recommendations according to the IV Brazilian Guidelines on Dyslipidemia and Atherosclerosis Prevention [27]. The guidelines were as follows: hold three meals and two snacks throughout the day and do not skip meals; include daily six portions of the group of cereals, tubers, and roots, preferring whole foods and in their most natural form; eat three servings of vegetables daily, as well as at least three servings of fruit for dessert or snacks; eat at least five times a week beans with rice because of their complete protein combination; consume three servings of milk and dairy products daily and a portion of meat, poultry, fish, or eggs, as well as removing the apparent fats from meat and poultry skin prior to preparation; ingest at most a portion of vegetable oil, olive oil, butter, or margarine; avoid soft drinks or industrialized juices, cakes, sweet and stuffed biscuits, desserts, and other goodies; decrease the amount of salt in the food and remove the salt shaker from the table; drink at least two liters of water daily at meal intervals. Practice thirty minutes daily of physical activity, avoiding alcohol and smoking. The macronutrient and micronutrient guidelines are contained in Table 1. Individuals in the S group included 14 g of *in natura* GB on meals. The choice of the amount of GB administered was based on a previous study conducted by Bucheli et al. [28] in volunteers between 65 and 70 years. In that study, Bucheli et al. showed positive effects in the use of GB. The authors concluded that dietary supplementation of goji berries for 30–90 days can prevent or reduce damage caused by free radicals by increasing antioxidant capacity in both 30 and 90 days, compared to the placebo group. Daily dietary supplementation with GB for 30–90 days increases plasma zeaxanthin and total antioxidant levels as well as protects from hypopigmentation and accumulation of soft drusen in the macula of elderly subjects [28]. Constituents of GB that have biological effect are zeaxanthin, rutin, betaine, cerebroside, *β*-sitosterol, flavonoids, amino acids, minerals, vitamins (in particular, riboflavin, thiamin, and ascorbic acid), and quercetin in addition to other phenolic compounds [21, 22]. The patients were passed through nutritional status and clinical, anthropometric, and biochemical evaluation at the beginning (baseline), 15 and 45 days after the supplementation had started.

### 2.3. Assessment of Anthropometric Measures

The anthropometric parameters, weight [29], height [29], and waist circumference [30, 31], were measured by a properly trained person, according to previously established criteria. The evaluation of the nutritional status was obtained by calculating the body mass index (BMI) [29]. The elderly ones had the nutritional status evaluated by BMI using the cutoff points defined for this age group [32].

### 2.4. Blood Sampling

Venous blood samples (20 ml) were obtained from participants after overnight fasting between 7:00 and 9:00 am. The samples were divided into three aliquots, in clot activator tube, EDTA (ethylenediaminetetraacetic acid), and sodium fluoride tubes in order to biochemical analyses and determination of redox state markers. Serum and plasma samples were obtained after centrifuging at 300*g* for 15 min at 4°C and were stored at −80°C until analyses.

### 2.5. Biochemical Assays

The serum concentrations of total cholesterol (TC) and triglycerides (TG) were determined by automated and colorimetric methods (Trinder's reaction; Labtest, Lagoa Santa, MG), and the HDL-C density was determined by homogeneous method (Labtest, Lagoa Santa, MG). LDL-C was estimated by the Friedewald equation (LDL-C = CT − (HDL-C + TG/5)) [33] or quantified by the homogeneous method (Labtest, MG) when the participant presented values of TG equal to or above 400 mg/dl. The LDL-C fraction was determined by the homogeneous LDL-C reagent (Labtest, Lagoa Santa, MG) after the selective precipitation of the other lipoproteins with heparin and magnesium, according to the procedure previously described by Hirano et al. [34] (VLDL), calculated by subtracting LDL and HDL from total cholesterol, and was determined by the values of serum cholesterol (Roche Diagnostics, Basel, BS, Switzerland). All measurements were carried out on the Cobas Mira Plus® automated equipment (Roche Diagnostics, Basel, BS, Switzerland). The concentrations of glucose (oxidase/peroxidase) were determined in automated equipment (Cobas Mira Plus, Roche Diagnostics, Basel, Switzerland) according to the manufacturer's specifications of the Labtest, Lagoa Santa, MG, kit. Serum concentrations of transaminases aspartate aminotransferase (AST) and alanine aminotransferase (ALT) were determined by the continuous ultraviolet kinetic methods, easily adaptable to automatic analyzers of the Bioplus BIO 2000 brand. The methodology allows accurate and accurate results to be obtained. Quality control was performed with the PROEX biochemical controls of the National Quality Control Program (PNCQ), which is sponsored by the Brazilian Society of Clinical Analyzes (SBAC).

### 2.6. Oxidative Stress Biomarkers

Serum antioxidant capacity was determined according to the ferric reducing antioxidant potential (FRAP) assay as described by Benzie and Strain [35]. In this procedure, the antioxidants present in the serum were evaluated as reducers of Fe^3+^ to Fe^2+^, which is chelated by 2,4,6-tris(2-pyridyl)-s-triazine (TPTZ) to form a Fe^2+^–TPTZ complex with maximum absorbance at 593 nm. Ten microliters of serum were mixed with 1 mL of reagent containing 1.7 mM FeCl_3_ and 0.8 mM TPTZ, prepared in 300 mM sodium acetate (pH 3.6). The samples were incubated for 15 min at 37°C, and the absorbance was read at 593 nm (Bioplus BIO 2000, Barueri, SP, Brazil). The 6-hydroxy-2,5,7,8-tetramethylchroman-2-carboxylic acid (Trolox) was used as a standard, and the FRAP values were expressed as Trolox equivalents in micromoles per liter. Blood reduced glutathione (GSH) was assessed according to the method proposed by Beutler et al. [36]. An aliquot of the total EDTA-treated blood was hemolyzed with cold water, and the proteins were precipitated by the addition of 30% trichloroacetic acid. Aliquots of 50 mL of the hemolyzed sample and 50 mL of 10 mM 5,50-dithiobis-2-nitrobenzoic acid were mixed in tubes containing 0.8 mL of 200 mM phosphate buffer, pH 8.0. After 3 min, the absorbance of the thiolate anion was measured at 412 nm (Bioplus BIO 2000, Barueri, SP, Brazil). GSH was used as a standard, and the results were expressed in micromoles per liter. Lipid peroxidation was performed by the detection of the oxidation product derivatives, substances that react with thiobarbituric acid, with malondialdehyde (MDA) being the most important, according to the previously described procedure. The absorbance of the supernatant was read at 532 nm [37]. The activity of the antioxidant enzyme catalase (CAT) and superoxide dismutase (SOD) was quantified in erythrocytes. Aliquots of the blood were centrifuged (700 g, 4°C, and 10 min), and erythrocytes were separated from the plasma. An equal volume of physiological solution was added, and the tubes were homogenized and centrifuged (700 g, 4°C, and 10 min). This procedure was repeated three times. Then, the erythrocytes were mixed with a hemolyzing solution (4 nM magnesium sulfate and 1 nM acetic acid), and the hemolyzed solution was stored for a maximum of 10 days at −80°C for analysis. CAT activity was quantified by monitoring the H_2_O_2_ decomposition in a spectrophotometer for 2 min at 240 nm [38]; its results were expressed in mmol/mg protein/second. SOD activity was assessed by the SOD-WST-SIGMA kit which allows the assay to be made using a water-soluble tetrazolium salt, WST-1 (2-(4-iodophenyl)-3-(4-nitrophenyl)-5-(2,4-disulfophenyl)-2 (tetrazolium, monosodium salt), which produces a water-soluble formazan dye after reduction with a superoxide anion. The absorbance at 440 nm is proportional to the amount of the superoxide anion, the SOD activity was then measured by reducing the color at 440 nm, and the results were expressed as U/ml. Total proteins were determined according to a specific method, using bovine albumin as standard. Aliquots of the hemolyzed solution were added to the Bradford reagent and kept in the dark at room temperature for 5 min, and the absorbance reading was performed at 595 nm [39] with results expressed as *μ*g/*μ*l protein.

### 2.7. Inflammatory Biomarkers

C-reactive protein (CRP) levels were determined by the nephelometry technique on BNII analyzer (Dade Behring, Marburg, Germany). The reagent consists of a suspension of polystyrene particles, coated with anti-PCR monoclonal antibody, which agglutinate in the presence of CRP from the sample. The intensity of diffused light in the nephelometer depends on the CRP concentration of the sample, so that, in comparison with dilutions of a standard of known concentrations, it is possible to determine the concentration of this mediator in the samples. The limit of the technique is 0.175 mg/l [40]. BD OptEIA diagnostic kits (Franklin Lakes, New Jersey, USA) were used for the determination of tumor necrosis factor (TNF-*α*), monocyte chemotactic protein 1 (MCP-1), interleukin 6 (IL-6), and interleukin-1*β* (IL-1*β*). All diagnostic kits consist of sandwich-type ELISA (immunoenzyme assay) and were performed according to the protocols provided by the manufacturer. Concentration calculations were performed using the equation of the straight line of each cytokine, obtained from calibration curves. The sensitivity of the assays is 2 pg/ml for TNF-*α*, 0.8 pg/ml for IL-1*β*, 2.2 pg/ml for IL-6, and 1.0 pg/ml for MCP-1.

### 2.8. Statistical Analysis

Continuous variables are presented as a mean and standard error of the mean, and categorical variables are presented as absolute frequency. All quantitative variables were tested for the normality by means of the Shapiro-Wilk test before analyses. Between groups, comparisons were analyzed by unidirectional ANOVA followed by the Bonferroni post hoc test; values of *p* < 0.05 were considered statistically significant.

## 3. Results

### 3.1. Participant Characteristics

Fifty participants were included (Table 1). Sociodemographic data demonstrated that, regarding gender, a similar distribution among groups was observed (72% women and 28% men in the C group and 68% women and 32% men in the S group). The average age in the C group was 49 years and in the S group 52 years. According to nutritional status, a division between adult and elderly individuals, in both groups, was performed; there were no patients with eutrophy, and the results of overweight and obesity are shown in Table 2. Regarding the use of the drugs, there were a similarity of consumption in both groups, especially the angiotensin-converting enzyme inhibitor (ACEI), angiotensin receptor blockers II (ARBs), and statins (Table 2).

### 3.2. Anthropometric Characteristics

When comparing the studied groups against the anthropometry and indicators of central obesity, significant differences were observed in relation to waist circumference (WC). The mean values were higher in group C when compared to those in group S; an interesting factor was that the supplemented group differed statistically at baseline against after a 45-day supplementation, reducing an average of 6.3 cm in the abdominal region during the 45-day study (Table 3).

### 3.3. Biochemical Assays

**Figure 1:**
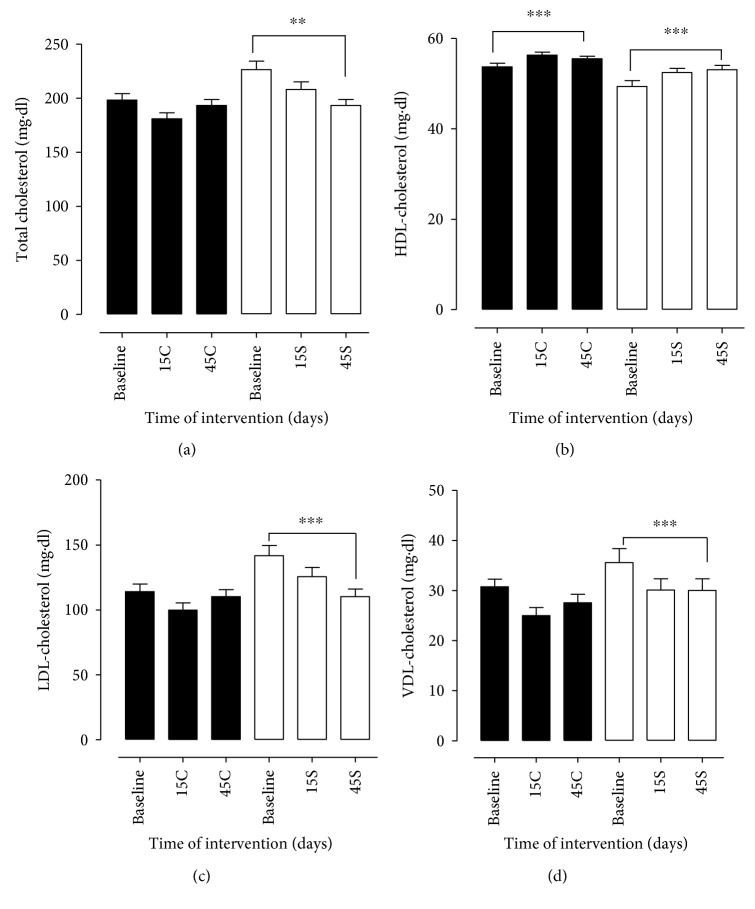
Lipid profile of patients with MS in the control group (C) and supplemented group (S) at the beginning, at day 15, and at day 45 after intervention. Total cholesterol (TC, (a)), HDL cholesterol (b), LDL cholesterol (c), and VLDL cholesterol (d) in patients with metabolic syndrome. Biochemical parameters were evaluated before (baseline) and 15 (15C and 15S) and 45 days (45C and 45S) after supplementation. Closed bars (control group—not supplemented) and open bars were supplemented with 14 g of goji berry daily. Values of ^∗∗^*p* < 0.01 and ^∗∗∗^*p* < 0.001 were considered statistically significant when comparing the baseline time with the end time of intervention in each group, using ANOVA followed by the Bonferroni post hoc test.

**Figure 2:**
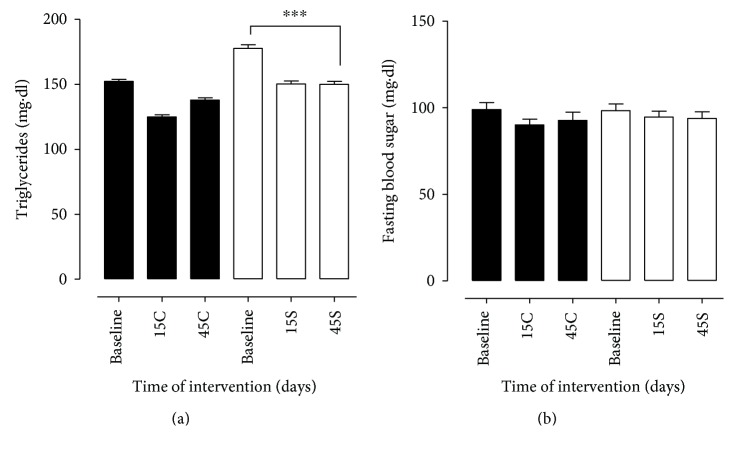
Serum triglycerides and fasting glycemia in patients with MS in the control group (C) and supplemented group (S) at the beginning, at day 15, and at day 45 after intervention. Evaluation of triglycerides (TG, (a)) and glucose (b) in patients with metabolic syndrome. Biochemical parameters were evaluated before (baseline) and 15 (15C and 15S) and 45 days (45C and 45S) after supplementation. Closed bars (control group—not supplemented) and open bars were supplemented with 14 g of goji berry daily. Values of ^∗∗∗^*p* < 0.001 were considered statistically significant when comparing the baseline time with the end time of intervention in each group, using ANOVA followed by the Bonferroni post hoc test.

**Figure 3:**
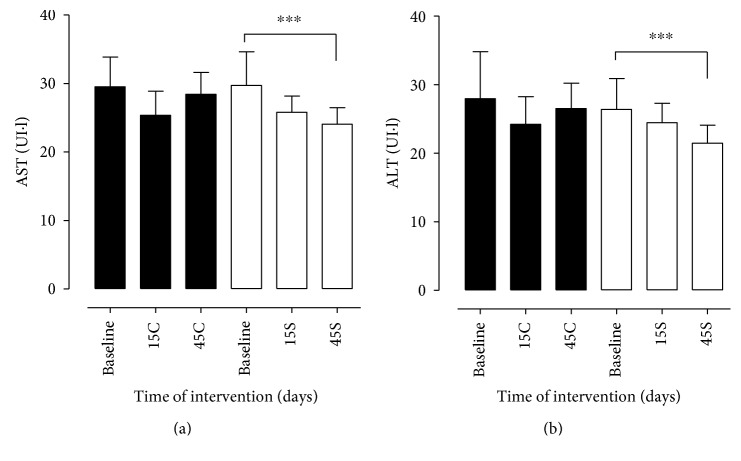
Hepatic enzyme patients with MS in the control group (C) and supplemented group (S) at the beginning, at day 15, and at day 45 after intervention. Evaluation of aspartate aminotransferase (AST) and alanine aminotransferase (ALT) in patients with metabolic syndrome. AST (a) and ALT (b) were evaluated before (group baseline) and 15 (15C and 15S) and 45 days (45S) after supplementation with 14 g of goji berry daily. The control group (closed bars) supplemented with goji (open bars). Values of ^∗∗∗^*p* < 0.001 were considered statistically significant when comparing the baseline time with the end time of intervention in each group, using ANOVA followed by the Bonferroni post hoc test.

Regarding the results of the laboratory data, in relation to total cholesterol, a significant reduction was observed in group S between baseline and 45 days of supplementation, which does not occur in group C, characterized by oscillation of the values between the times analyzed (Figure 1(a)). For HDL-C, the statistical difference indicated improvement in the S group over the evaluated times, unlike the C group, in which there was oscillation during the study period. Similarly, the results for LDL-C and VLDL-C were statistically significant for group S between baseline and 45 days, indicating a reduction in this indicator, thus corroborating for a positive result in the four lipid variables: TC, LDL-C, HDL-C, and VLDL-C. In group C, an oscillation was observed for LDL-C and VLDL-C among the analyzed times, as shown in Figure 1. In relation to serum levels of TG (Figure 2(a)), both groups differed significantly between baseline and 45 days, with a continuous and more significant reduction in group S. As for fasting blood sugar, the groups did not differ between themselves and between study times (Figure 2(b)). In this study, the plasma levels of AST and ALT were measured simultaneously, which presented similar trends. For AST and ALT values, groups C and S differed statistically between 45 days (*p* < 0.05 and *p* < 0.01, resp.). However, the improvement was observed in group S over the evaluated times (Figure 3).

### 3.4. Oxidative Stress Biomarkers

**Figure 4:**
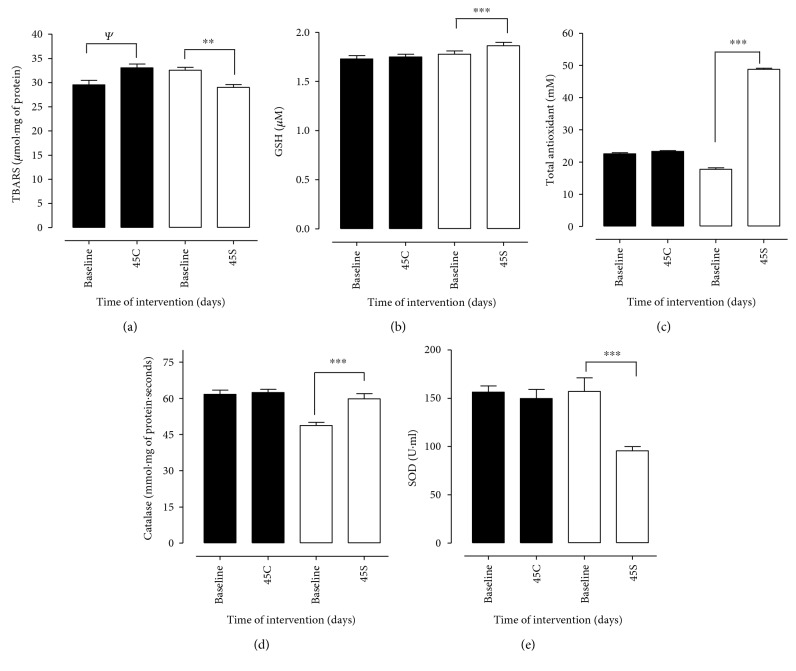
Oxidative stress variable patients with MS in the control group (C) and supplemented group (S) at the beginning and day 45 after intervention. Evaluation of thiobarbituric acid reactive substances (TBARS), blood reduced glutathione (GSH), total antioxidant control, catalase activity (CAT), and measurement of superoxide dismutase activity (SOD) in patients with metabolic syndrome. TBARS (a), GSH (b), total antioxidant control (c), and CzAT (d) were evaluated before (baseline) and 45 days after supplementation with 14 g of goji berry daily. Values of ^∗∗^*p* < 0.01 and ^∗∗∗^*p* < 0.001 were considered statistically significant when comparing the baseline time with the end time of intervention in each group, using ANOVA followed by the Bonferroni post hoc test.

In relation to oxidative stress analysis, plasmatic lipid peroxidation was evaluated by the TBARS method (thiobarbituric acid reactive substances) and a reduction of 15% in group S between baseline and after 45 days of supplementation and an increase of 11.9% in group C was observed, considering the same experiment period (Figure 4(a)). Significant results for GSH were found for group S at the baseline and after 45 days of supplementation, presenting a 5.1% increase in values, in contrast to the results found in group C that remained without oscillation at the beginning and at the end of the experiment (Figure 4(b)). For total antioxidant, group S presented statistically significant results, indicating an increase of 175.3% between the baseline and after 45 days of supplementation, in contrast to group C, which maintained the results at the beginning and end of the experiment (baseline = 22.54 mM and after 45 days = 23.27 mM) (Figure 4(c)). The activity of the CAT enzyme showed statistically significant results for the S group, which presented a 22.6% increase between baseline and after 45 days of supplementation, unidentified evidence for group C, in which the results remained similar during the study period (Figure 4(d)). The results for SOD indicated a significant reduction of 39.1% in group S, comparing the baseline and after 45 days of supplementation. In contrast to the results found for group C where there was a reduction of only 4.1% among the evaluated times (Figure 4(e)).

### 3.5. Inflammatory Biomarkers

Regarding the analysis of the inflammatory profile in this study, although CRP presented a good inflammatory parameter, in this sample, there was no relationship between the groups and SM (Table 4). Results regarding the MCP-1 and TNF-*α* variables showed that the values did not oscillate between the times analyzed in both groups (Table 4). For the analyses of interleukins IL-1*β* and IL-6, the sensitivity of the method (0.8 pg/ml and 2.2 pg/ml, resp.) used to verify was not able to identify its presence in the samples.

## 4. Discussion

This study demonstrated that daily ingestion of 14 g of goji berry, a fruit rich in phenolic antioxidants [20–22], for 45 days increased the antioxidant protection of plasma and whole blood, thereby lowering oxidative stress in individuals with MS. Associated with improvement in the redox state, a significant reduction in abdominal fat was observed as evidenced by the reduction in waist circumference with an improvement in the lipid profile of the volunteers who received daily GB. It could be affirmed that the observed effects are due to the introduction in the diet of GB since both groups were composed of volunteers with MS and that they received the same orientation of diet based on recommendations according to the IV Brazilian Guidelines on Dyslipidemia and Atherosclerosis Prevention [27].

When analyzing the demographic data of the current study, it was observed that there is a predominance of females. Data from the National Cholesterol Education Program Adult Treatment Panel III (NCEP-ATP III) also show that 60% of women and 45% of North American men have a diagnosis of MS [41]. The results corroborate with other findings from Brazilian studies with a higher prevalence of MS in the female population [42–44].

The prevalence of overweight and obesity in all study participants support the relationship between subcutaneous adipose tissue and MS, and it is consistent with the literature. A research through computed tomography performed on 365 individuals, 187 patients with MS, and 187 patients without MS indicated that subcutaneous adipose tissue was significantly associated with MS in men and women, and it was also associated with inflammation increase and oxidative stress [45].

Corroborating the data cited in this study on waist circumference reduction, a study investigating the effects of quercetin that is found in onions (*Allium cepa* L.) on lipid profile and antioxidant status in moderately hypercholesterolemic individuals indicated that onion juice decreased significantly waist circumference, total cholesterol, and LDL-C. In addition, it increased the total antioxidant capacity, being recommended to combat cardiovascular diseases [46].

The data found in this study supports the hypothesis that the antioxidant effects of GB influenced the reduction of WC. The antioxidant action was also observed in a study of orange juice ingestion in different concentrations of polyphenols (299 and 745 mg/day) on the antioxidant defense system, oxidative stress, biomarkers, and clinical signs of MS in 100 individuals. A reduction in BMI, WC, and leptin was observed in the results, indicating the benefits of phenolic compounds [16] similar to those found in the present study with GB.

In relation to the lipid profile, in the supplemented groups with GB, the reduction of TG was sharper. Similar results were found in fish oil (3 g) research, rich in polyunsaturated fatty acids, indicating a reduction of TG in the group that used fish oil when compared to the control group (without substance use). In addition to this indicator, there was an increase in total antioxidant capacity [47], a fact that was also observed in our study with GB supplementation. The reduction in TG levels observed in the C group is probably due to the introduction in the diet of a greater quantity of fruits and vegetables since all the volunteers were directed to introduce a healthy diet as described in Materials and Methods.

The results presented in Figure 2(b) indicate that supplementation with GB was not able to improve the glycemic levels of the volunteers. However, it is observed that in both groups, the volunteers present glycemic values close to the reference values; this fact is probably due to the use of oral hypoglycemic agents since 100% of the volunteers are users of these drugs.

The findings of the current study indicate an improvement in serum levels or transaminases (ALT and AST) in the group supplemented with GB. Similar results were found in a study that investigated the effects of GB on alcohol-induced hepatic disease, and the authors found that the administration of this compound significantly inhibited the increase in serum AST and ALT activity caused by ethanol ingestion, and also, an increase in antioxidant protection (GSH, CAT, SOD, and GPx) and a reduction in lipid peroxidation were identified [48]. It could be inferred that the reduction in transaminase levels is associated with GB supplementation since group C shows no change in serum transaminase levels. Results like these demonstrate that the GB is a promising agent to protect the liver from hepatotoxicity and accumulation of liver fat.

In addition, evaluating hepatotoxicity, an experimental study with rats indicates that GB offers protection against acute hepatotoxicity caused by the drug paracetamol. Two groups were studied, one of which received a dose of 100 mg/kg of GB extract dissolved in physiological solution intraperitoneally for 7 days. After 7 days, a single dose of paracetamol (1 g/kg) was given to both groups. The serum markers of AST, ALT, total antioxidant capacity, and total oxidizing state were evaluated after 24 hours, and the group supplemented with GB presented better marker results [49].

The antioxidant/oxidative stress status in the evaluated groups (C and S) showed that in the S group, there was a significant reduction in the lipoperoxidation values evaluated by the plasma levels of TBARS (Figure 4(a)). The reduction of TBARS values in group S is probably associated with the consumption of GB, since associated with this a significant increase was observed in total antioxidant values in group S. Studies that evaluated the anti-inflammatory and antioxidant effects of GB, *Vaccinium marcrocarpon* (cranberry), and *Vaccinium myrtillus* (blueberry) extracts in rats showed a reduction in TBARS levels in all tested groups [21], a result that corroborates with the one found in this research with the supplementation of GB *in natura*.

Elevated levels of oxidative stress and adipokines are found in obese patients, associated with lower glutathione levels. In the present study, there was a significant increase in the GSH values in the S group after 45 days of supplementation, indicating an improvement in the oxidative profile. Similar results were found in another study, in which hepatic GSH values were significantly higher in animals treated with goji berry extracts than with cranberry [50].

The goji berry used in the study was able to significantly increase the total antioxidant activity in the supplemented group when compared to that in the control group (Figure 4(c)). The supplementation with antioxidants has the function of reducing oxidative stress because all nucleated cells respond to stress by positively regulating a complex set of defense mechanisms, a result observed in the present study through the daily insertion of GB into the diet.

Corroborating with the results of this study, a study conducted to evaluate the effects of vitamin C supplementation and antioxidant defenses presented data indicating that such supplementation causes a baseline increase in the expression of these defenses and modifies the responses of both muscle cells and lymphocytes to oxidative stress [51]. Similar results were found in the research using a combination of supplements with vitamins A, C, and E over a period of 8 weeks, having, as a result, the reduction of oxidative stress and indicating a potential mechanism underlying the protection of cardiovascular health and diabetes. Evidence from these studies suggests that obesity directly influences the early development of insulin resistance and endothelial dysfunction, and it can be beneficially influenced by the ingestion of antioxidant foods [52].

Similar results were found for individuals with a high risk of developing cardiovascular diseases or dyslipidemias and human embryonic kidney-293 (HEK-293) cells. This effect were found after an increased consumption of foods rich in fiber, fruits, vegetables, or mate tea [14, 53, 54]. Our results corroborate with these studies since we observed an increase in the total antioxidant potential in volunteers who introduced 14 g of GB for 45 days (Figure 4(c)).

Studies have also shown that phenolic compounds from various sources are effective in improving antioxidant enzymes, suggesting their role in the prevention and treatment of oxidative stress-related diseases. Hence, the consumption of phenolic-rich fruits has been associated with reduced levels of ROS in animal experiments [55, 56]. Likewise, a high intake of phenolic-rich fruits has been reported to enhance the activities of the antioxidant enzymes [57].

Although studies have reported an increase in the activity of antioxidant enzymes in individuals who consume foods rich in phenolic compounds, our results show an increase in CAT activity and a significant reduction in SOD activity (Figures 4(d) and 4(e)). These findings may be related to the effect of quercetin, zeaxanthin, and rutin, the main phenolic constituents of GB [20–22] since studies report that the antioxidant effect of phenolic compounds could be associated with prevention in the formation of reactive species or in the improvement of enzyme activity antioxidants [58].

This decrease in SOD activity may be associated primarily with the antioxidant components of GB that may act directly preventing the formation of the superoxide anion radical since some studies show that antioxidant-rich fruit extracts are able to increase the expression of mRNA to CAT without changing the mRNA expression for SOD [10, 14]. In previous studies, we showed that the extract of GB presents high scavenging capacity against the DPPH radical; this shows its ability to act as a scavenger of reactive species [21], and this could explain the reduction of SOD activity in the supplemented group. In addition, the reduction of SOD activity may be explained by the ability of GB to increase other SOD isoforms such as Mn-SOD (SOD2) found in the mitochondrial matrix or Fe-SOD (SOD3) found in the extracellular medium [58]. In our study, we determined only the CuZn-SOD isoform (SOD1), which is found in erythrocytes.

In assessing the results of the inflammatory profile, despite CRP, MCP-1, and TNF-*α* presented as good inflammatory parameters, in this study, there was no relationship observed between the groups and MS during the evaluated period. For the analyses of IL-1*β* and IL-6, the sensitivity of the method used to verify was not able to identify its presence in the samples. These findings may be associated with the medications used by both groups (C and S) since all patients use oral hypoglycemic agents (metformin and glibenclamide) and statins. Studies show that metformin, glibenclamide, and statins reduce the production of the inflammatory cytokine IL-1*β* and CRP and the expression of TNF-*α* [59–61].

## 5. Conclusion

The results of this study demonstrated an increase in serum antioxidant capacity and GSH and a decrease in lipid peroxidation, LDL cholesterol, and waist circumference after long-term ingestion of GB, suggesting that this is an effective dietary supplement for the prevention of cardiovascular diseases in individuals with MS following a free diet.

## Figures and Tables

**Table 1:** Guidelines related to the amount of macronutrients and micronutrients of both groups participating in the research.

Food group	Amount of servings per day	Average caloric value per serving
Rice, bread, pasta, potato, and cassava	6	150 kcal
Vegetables	3	15 kcal
Fruits	3	70 kcal
Beans	1	55 kcal
Milk, cheese, and yogurt	3	120 kcal
Meat, fish, and eggs	1	190 kcal
Oil and fat	1	73 kcal
Sugar and candies	1	110 kcal

**Table 2:** Sociodemographic, anthropometric, and drug use characteristics in patients with metabolic syndrome, belonging to the control group (C) and supplemented group (S).

Variables	Groups	
	C (*n* = 25)	S (*n* = 25)
	Mean ± SE	Mean ± SE
Age (years)	49.17 ± 2.56	52.6 ± 2.24

Age group^∗^		
Up to 59 years	20 (80%)	19 (76%)
60 years and over	5 (20%)	6 (24%)

Gender^∗^		
Female	18 (72%)	17 (68%)
Male	7 (28%)	8 (32%)
BMI (kg/m^2^)	33.72 ± 1.28	32.98 ± 0.71

Adults^∗^		
Eutrophy	—	—
Overweight	7 (33.3%)	5 (21.7%)
Obesity	14 (66.5%)	16 (73.8%)

Elderly^∗^		
Eutrophy	—	—
Overweight	2 (50%)	2 (66.6%)
Obesity	2 (50%)	1 (33.3%)
Systolic blood pressure (mmHg)	128 ± 6	131.6 ± 3
Diastolic blood pressure (mmHg)	82 ± 3	84.8 ± 2

Drugs in use^∗^		
ACEI/ARBs	20 (80%)	21 (84%)
Statins	13 (52%)	12 (48%)
Oral hypoglyclers	9 (36%)	7 (28%)
Diuretics	8 (32%)	7 (28%)

^∗^Outcome presented in the form of *n* (%). SE: standard error; ACEI: angiotensin-converting enzyme inhibitor; ARBs: angiotensin receptor blockers II.

**Table 3:** Anthropometric characteristics in patients with metabolic syndrome, belonging to the control group (C) and supplemented group (S).

Variables	Groups					
	C (*n* = 25)			S (*n* = 25)		
	Mean ± SE			Mean ± SE		
	Time of intervention (days)			Time of intervention (days)		
	Baseline	15	45	Baseline	15	45
Anthropometric characteristics						
Weight (kg)	91.59 ± 23.92	92.10 ± 24.11	92.43 ± 24.07	87.56 ± 15.42	87.24 ± 15.31	87.29 ± 15.34
BMI (kg/m^2^)	33.72 ± 1.28	33.9 ± 1.29	34.03 ± 1.29	32.98 ± 0.71	32.87 ± 0.70	32.84 ± 0.70
WC (cm)	104.4 ± 16.92	104.14 ± 16.66	104.64 ± 17.34	106.46 ± 9.78	103.66 ± 9.69	100.08 ± 19.78^∗∗∗^

Values are expressed as mean ± SEM (mean standard error). ^∗∗∗^*p* < 0.01: significant difference between baseline and 45 days in the supplemented group.

**Table 4:** Inflammatory profile of patients with metabolic syndrome, belonging to the control (C) and supplemented (S) group, before and after the use of 14 g goji berry daily.

	Groups			
Variables	C (*n* = 25)		S (*n* = 25)	
	Mean ± SE		Mean ± SE	
	Time of intervention (days)			
Inflammatory profile	Baseline	45	Baseline	45
TNF-*α* (pg/ml)	38.88 ± 9.71	20.85 ± 4.41	18.98 ± 3.50	16.50 ± 1.47
MCP-1 (pg/ml)	21.09 ± 2.21	21.29 ± 3.16	16.44 ± 0.79	16.14 ± 1.00
CRP	4.66 ± 0.55	3.94 ± 0.41	3.72 ± 0.44	4.04 ± 0.48

Values are expressed as mean ± SEM (mean standard error).

## Abbreviations

ACEI: Angiotensin-converting-enzyme inhibitor
ALT: Alanine aminotransferase
ARBs: Angiotensin receptor blockers II
AST: Aspartate aminotransferase
BMI: Body mass index
CAT: Catalase
CRP: C-reactive protein
CVD: Cardiovascular disease
DBP: Diastolic blood pressure
DM2: Diabetes mellitus type 2
DNA: Deoxyribonucleic acid
DPPH: 2,2-diphenyl-1-picrylhydrazyl
EDTA: Ethylenediaminetetraacetic acid
FRAP: Ferric reducing antioxidant potential
GB: Goji berry
GPx: Glutathione peroxidase
GSH: Reduced glutathione
HDL: High-density lipoprotein
IL-1*β*: Interleukin 1 beta
IL-6: Interleukin 6
IL-18: Interleukin 18
LDL: Low-density lipoprotein
MCP-1: Monocyte chemotactic protein
MDA: Malondialdehyde
MS: Metabolic syndrome
RNS: Reactive nitrogen species
ROS: Reactive oxygen species
SBP: Systolic blood pressure
SOD: Superoxide dismutase
TBARS: Thiobarbituric acid reactive substances
TC: Total cholesterol
TG: Triglycerides
TNF-*α*: Tumor necrosis factor alpha
VLDL: Very low-density lipoprotein
WC: Waist circumference.
